# Enhancing immunotherapy outcomes by targeted remodeling of the tumor microenvironment via combined cGAS-STING pathway strategies

**DOI:** 10.3389/fimmu.2024.1399926

**Published:** 2024-05-16

**Authors:** Mingqing Huang, Zhuocen Cha, Rui Liu, Mengping Lin, Naif Abdul Gafoor, Tong Kong, Fei Ge, Wenlin Chen

**Affiliations:** ^1^ Third Department of Breast Surgery, The Third Affiliated Hospital of Kunming Medical University, Yunnan Cancer Hospital, Kunming, China; ^2^ Guizhou Hospital of the First Affiliated Hospital, Sun Yat-sen University, Guizhou, China; ^3^ Department of Breast Surgery, The Third Affiliated Hospital of Kunming Medical University, Yunnan Cancer Hospital, Kunming, China; ^4^ International Education School of Kunming Medical University, Kunming, China; ^5^ Department of Gynecology, The Third Affiliated Hospital of Kunming Medical University, Yunnan Cancer Hospital, Kunming, China; ^6^ Department of Breast Surgery, The First Affiliated Hospital of Kunming Medical University, Kunming, China

**Keywords:** cGAS-STING, hot tumor, tumor immunology, immune checkpoint inhibitor (ICI), tumor microenvironment

## Abstract

Immune checkpoint inhibitors (ICIs) represent a groundbreaking advance in the treatment of malignancies such as melanoma and non-small cell lung cancer, showcasing substantial therapeutic benefits. Nonetheless, the efficacy of ICIs is limited to a small subset of patients, primarily benefiting those with “hot” tumors characterized by significant immune infiltration. The challenge of converting “cold” tumors, which exhibit minimal immune activity, into “hot” tumors to enhance their responsiveness to ICIs is a critical and complex area of current research. Central to this endeavor is the activation of the cGAS-STING pathway, a pivotal nexus between innate and adaptive immunity. This pathway’s activation promotes the production of type I interferon (IFN) and the recruitment of CD8^+^ T cells, thereby transforming the tumor microenvironment (TME) from “cold” to “hot”. This review comprehensively explores the cGAS-STING pathway’s role in reconditioning the TME, detailing the underlying mechanisms of innate and adaptive immunity and highlighting the contributions of various immune cells to tumor immunity. Furthermore, we delve into the latest clinical research on STING agonists and their potential in combination therapies, targeting this pathway. The discussion concludes with an examination of the challenges facing the advancement of promising STING agonists in clinical trials and the pressing issues within the cGAS-STING signaling pathway research.

## 1 Introduction

Tumor initiation, progression, and metastasis are intimately linked to the multifaceted tumor microenvironment (TME) (1). This network, enveloping the tumor within the body, comprises tumor cells and a variety of immune cells including T and B lymphocytes, dendritic cells (DCs), natural killer (NK) cells, and tumor-associated macrophages (TAMs), as well as stromal cells such as tumor-associated fibroblasts, mesenchymal stromal cells, and endothelial cells (2, 3). These cells engage in intricate interactions through signaling pathways, secreting a myriad of growth factors, cytokines, chemokines, and other biological factors, thereby fostering a complex tumor microecology that dictates the rapid proliferation and diversification of tumor cells (4). Thus, the tumor and its microenvironment are co-dependent, with their relationship characterized by mutual reinforcement as well as antagonism. The phenomenon of tumor immune escape is intricately associated with the TME’s heterogeneity, potentially affecting the responsiveness to immunotherapy (5, 6).

**Figure 1:**
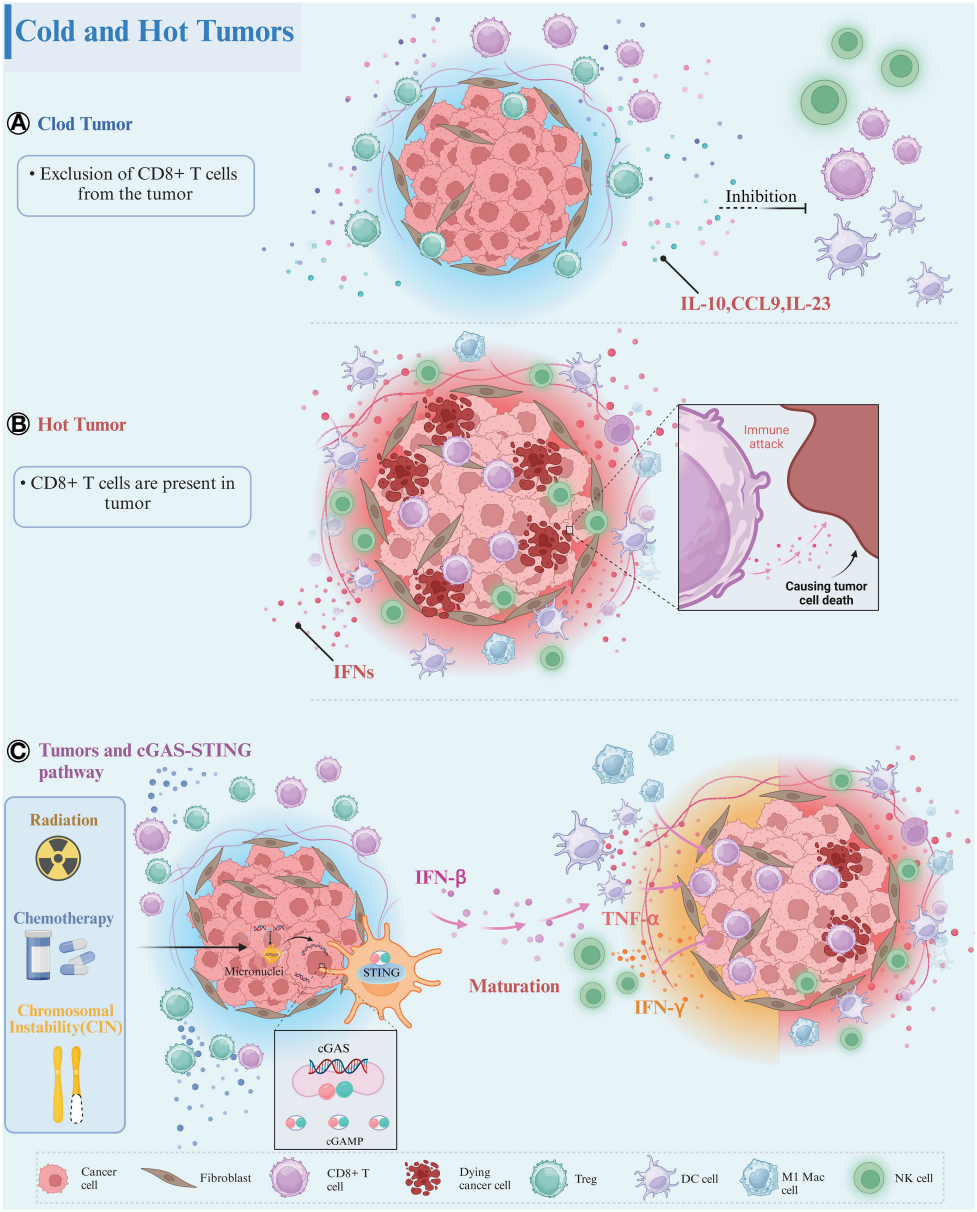
Tumor immune phenotypes. **(A)** Cold Tumors: In cold tumors, the absence of CD8^+^ T cells within the tumor parenchyma or its vicinity is remarkable. These tumors foster an immunosuppressive microenvironment characterized by the presence of immune-inhibitory cell populations, such as Treg cells. These cells secrete various immunosuppressive factors, including IL-10, CCL9, and IL-23, which impede the maturation of immune cells and contribute to the establishment of an immunosuppressive milieu conducive to tumor progression. **(B)** Hot Tumors: Conversely, in hot tumors, infiltration of CD8^+^ T cells into the tumor parenchyma is evident. These tumors display robust interferon signaling in the peritumoral region and harbor a diverse array of mature immune cells within the tumor parenchyma. **(C)** Tumors and cGAS-STING Signaling Pathway: CIN as well as radiotherapy/chemotherapy can induce nuclear DNA breaks in tumor cells, activating the cGAS-STING signaling pathway to produce large amounts of IFN-β. IFN-β can mature and activate a variety of immune cells, including DCs, NK cells, M1 macrophages, induce cytokine release, and recruit more CD8^+^ T cells into the tumor parenchyma. The crosstalk between cold and hot tumors is mediated through the engagement of the cGAS-STING signaling axis. Created with BioRender.com.

Based on the spatial distribution of CD8^+^ cytotoxic T lymphocytes (CTLs) within the TME, tumors have been categorized into three primary immune phenotypes: immune-inflamed, immune-excluded, and immune-desert (7, 8). In immune-inflamed tumors, CD8^+^ T cells penetrate the tumor parenchyma, whereas in immune-excluded tumors, they gather around the tumor parenchyma without infiltrating it, and are completely absent in immune-desert tumors and their peripheries (9, 10). Immune-inflamed tumors, termed “hot” tumors, demonstrate a notable response to treatment with immune checkpoint inhibitors (ICIs) (11, 12), characterized by significant T cell infiltration, increased inflammatory signaling (notably interferon-γ), heightened PD-L1 expression, and a substantial mutational burden (11, 13). Conversely, “cold” tumors, which include immune-excluded and immune-desert variants, are marked by a scant tumor mutational load, diminished expression of major histocompatibility complex (MHC)-class I molecules, decreased PD-L1 expression, and inadequate T cell infiltration (11, 13). Additionally, the presence of immunosuppressive cells such as myeloid-derived suppressor cells (MDSCs), TAMs, and regulatory T cells (Tregs) within cold tumors contributes to tumor immune evasion, obstructing the activation, proliferation, and cytotoxic function of CD8^+^ T cells (13) (**Figure 1**). Tumors with limited antigenic diversity and minimal inflammation are inherently more resistant to immunotherapy, attributed to their lack of innate and adaptive immune characteristics (7).

Immunosurveillance, the immune system’s capacity to detect, eliminate, and swiftly clear mutant cells, acts as a critical defense against tumor development. The ability to circumvent immune surveillance is essential for tumor progression, as malignant tumors evolve in the shadow of immune vigilance (14, 15). Mechanisms facilitating immune evasion include tumor antigen loss, resistance to apoptosis, antigenic modulation, restricted expression of MHC class I molecules, aberrant costimulatory signaling, and the recruitment of inhibitory immune cells by tumor cells to secrete immunosuppressive factors (16). The innate immune response serves as the primary defense mechanism against emerging tumors, while adaptive immune responses deliver more targeted effects via cytotoxic T lymphocytes and TH1 cells (14, 17). ICIs target inhibitory signals from receptors such as PD-1 or CTLA-4, commonly exploited by cancer cells on tumor-infiltrating lymphocytes (TILs), thereby restoring CTL-mediated immunity (18, 19).

**Figure 2:**
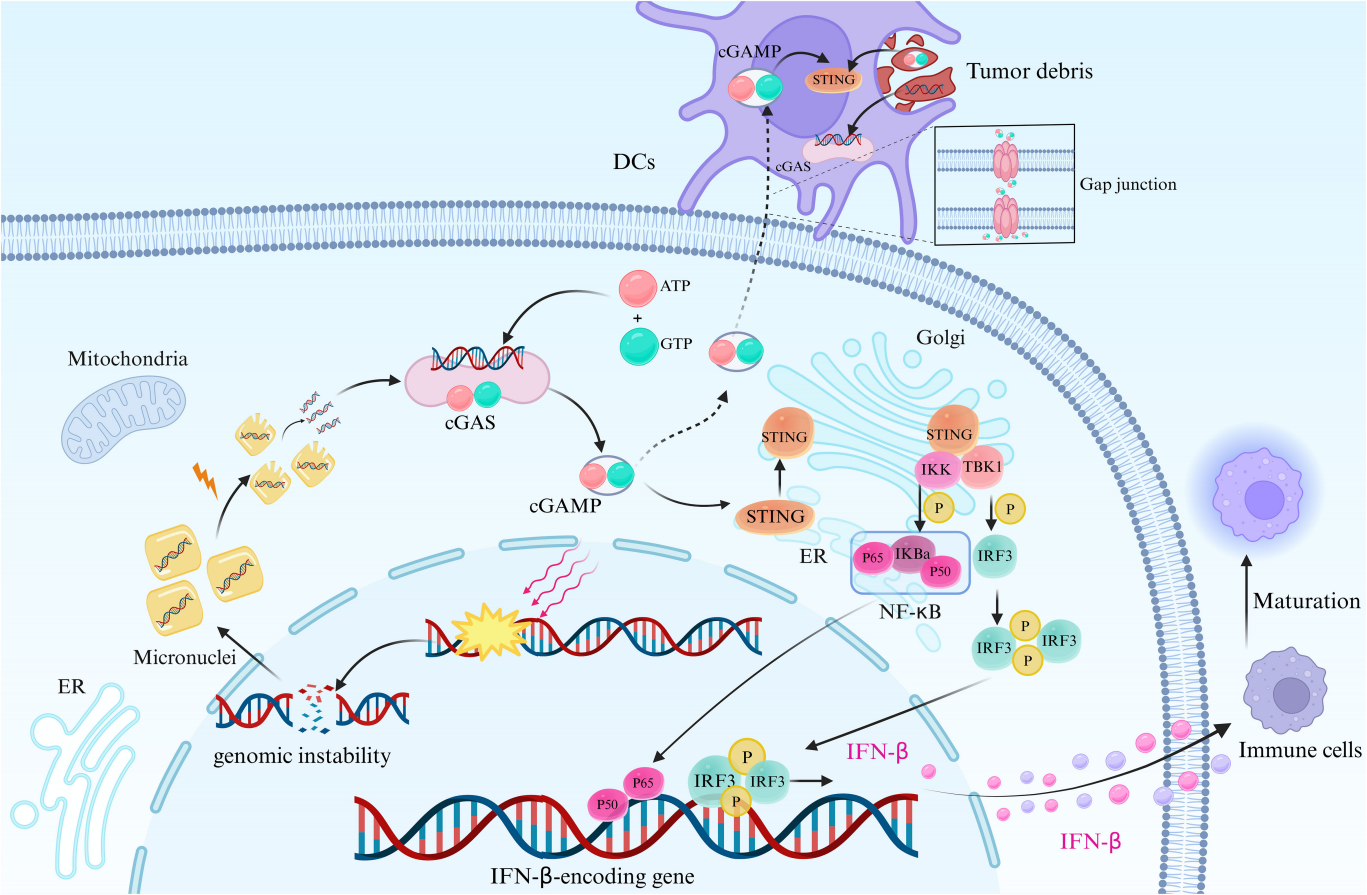
cGAS-STING signaling pathway in tumor cells. This figure depicts the cGAS-STING signaling pathway in tumor cells, initiated by genomic instability or DNA repair failure. This leads to nuclear DNA leakage and the formation of extranuclear micronuclei, facilitating the recognition of cytoplasmic DNA by cGAS upon micronucleus rupture. Subsequently, conformational changes occur in cGAS, and the synthesis of cGAMP is catalyzed by ATP and GTP. cGAMP then binds to STING, activating its transfer from the ER to the Golgi apparatus. This recruitment of STING leads to the binding of TBK1, which phosphorylates the transcription factor IRF3 (33). Phosphorylated IRF3 forms dimers that translocate into the nucleus, where they regulate the expression of IFN-β. Additionally, STING activation leads to the phosphorylation of IKK kinase, resulting in the release of NF-κB, which translocates into the nucleus. This induces the expression of IFN-β and inflammatory cytokines, along with IRF3 and other transcription factors. The synthesis and release of IFN-β stimulate the maturation of innate immune cells and promote the formation of the tumor inflammatory microenvironment. DCs possess the capacity to engulf cGAMP originating from tumor cells or neighboring cells, facilitated through mechanisms like intercellular communication via gap junctions and phagocytosis of tumor debris, which contains dsDNA as well. Following internalization, cGAMP activates the intracellular STING signaling pathway within DCs, prompting the production of IFN-β. Created with BioRender.com.

The Stimulator of Interferon Genes (STING) pathway, alongside cyclic guanosine-adenosine triphosphate synthase (cGAS), is widely expressed across various cell types, including immune, non-immune, and cancer cells (20–22). STING, functioning as one of the cytoplasmic DNA sensors, and cGAS, its upstream molecule, form a 2:2 complex upon DNA binding, triggering a conformational change and leading to the synthesis of cyclic GMP-AMP (cGAMP) through a process catalyzed by ATP and GTP (23–28). This cGAMP, serving as a second messenger, binds to STING on endoplasmic reticulum (ER) membranes, inducing conformational alterations in STING and the formation of oligomers (29–31). Upon activation, STING is translocated from the ER to the Golgi apparatus, where two cysteines (Cys88 and Cys91) undergo palmitoylation, facilitating the recruitment and binding of the kinase TANK-binding kinase 1 (TBK1), subsequently phosphorylating the transcription factor IRF3 (32). Phosphorylated IRF3 dimerizes and translocates into the nucleus, regulating the expression of type I interferon-beta (IFN-β) (**Figure 2**). Additionally, STING activates IKK kinases (TBK1 and IKKϵ), phosphorylating the IκB family, thereby releasing NF-κB, resulting in the expression of IFN and inflammatory cytokines (34–40). The cGAS-STING signaling pathway serves as the primary effector for cells to sense and respond to cytoplasmic abnormal double-stranded DNA (dsDNA), establishing an innate immune response with high efficiency by stimulating the expression and secretion of type I IFNs and interferon-stimulated genes, crucial components of host defense mechanisms in organisms (41, 42). Chromosomal segregation errors during mitosis lead to chromosomal instability (CIN), a phenomenon exacerbated by radiotherapy or chemotherapy, resulting in DNA double-strand breaks (DSBs) and the formation of extranuclear micronuclei within cancer cells (43). This persistent accumulation of cytosolic DNA has the potential to activate the cGAS-STING pathway (44–46).

STING-deficient mice have demonstrated increased susceptibility to various malignancies and reduced survival rates, underscoring the essential role of a robust STING-mediated immune response in tumor suppression (47–50). Type I IFNs significantly contribute to restraining tumor growth and extending host survival rates by mounting a specific immune response against tumors (51). Cancer cells expressing cGAS poses the ability to recognize cytoplasmic DNA and generate cGAMP, which in turn stimulates the secretion of IFN-β and tumor necrosis factor alpha (TNF-α) through the STING pathway (17). Simultaneous stimulation of IFN-β and TNF-α signaling induces significant necrosis of tumor cells (52). Numerous research studies have linked a poor prognosis in human cancers to the inhibition of the cGAS-STING pathway, as the lack of cytosolic DNA detection contributes to immune escape in cancer cells (53). The p53-p21 pathway facilitates cGAS-STING to promote cancer cell senescence (54), while cGAS-STING-mediated autophagy during a mitotic crisis in normal cells prevents their transformation into cancer cells, highlighting the pathway’s role in cellular homeostasis (55). To prevent an excessive response to the cGAS-STING pathway, cGAS-STING is digested in autophagy lysosomes following transient activation of downstream signaling (56).

In summary, enhancing tumor antigen specificity and immune cell effector functions is paramount for improving tumor immunity. This review focuses on the molecular mechanism of the cGAS-STING signaling pathway, analyzing its relationship to tumor immunity, and discussing how the innate immune pathway cGAS-STING transforms “cold” tumors into “hot” tumors, thereby further enhancing the therapeutic effect of ICIs.

## 2 Mechanisms of cGAS-STING-mediated antitumor effects

### 2.1 cGAS-STING: a nexus of innate and adaptive immunity

Innate immunity and adaptive immunity represent two interrelated subtypes of the immune response. The latter encompasses humoral and cell-mediated immunity, orchestrating the self-activation, proliferation, and differentiation of T and B lymphocytes into effector cells upon antigenic stimulation *in vivo*, thereby instigating a cascade of biological events, including antigen clearance, among others (57). Innate immunity, evolving over the course of evolutionary history, serves as the body’s initial defense against pathogenic intrusion, comprising monocytes/macrophages, DCs, granulocytes, NK cells, and NKT cells (58, 59).

The activation of the cGAS-STING pathway serves as a pivotal link between innate and adaptive immunity. Upon activation, this pathway induces the production of type I IFN, which, in conjunction with STING, has been demonstrated to foster the development of innate immune cells such as DCs and NK cells (60, 61), thereby facilitating adaptive immune responses against tumors. These findings underscore the crucial role of the host STING pathway in the innate immune recognition of immunogenic tumors. This cascade of events culminates in the activation of antigen-presenting cells (APCs), the production of IFN-β, and the initiation of CD8^+^ T cell responses against tumor antigens (48). TAMs represent a critical class of tumor-infiltrating immune cells intricately involved in tumor growth and metastasis by modulating tumor immune surveillance. Within this spectrum, M1 and M2 macrophages delineate two opposing functional continua, representing antitumor and immunosuppressive phenotypes, respectively (62). Recent studies have elucidated the role of the cGAS-STING signaling pathway in orchestrating TAM polarization towards the M1 phenotype (63, 64).

This pathway not only fosters the secretion of inflammatory factors and chemokines but also facilitates the recruitment and activation of T lymphocytes. Moreover, it promotes the differentiation of both CD4^+^ and CD8^+^ T cells, thereby establishing a robust connection between innate and adaptive immunity (62).

### 2.2 The role of type I IFNs in antitumor immunity

Type I IFNs play a pivotal role in fostering protective antitumor immunity, particularly in the context of immunogenic or “hot” tumors characterized by heightened T lymphocyte infiltration and improved response to ICIs compared to “cold” tumors (65, 66). These IFNs, downstream products of the cGAS-STING pathway, exert multifaceted effects on both innate and adaptive immunity.

Upon activation, type I IFNs facilitate the maturation of macrophages, NK cells (67, 68) and DCs (69). They orchestrate a cascade of events that encompass direct suppression of tumor cells and indirect antitumor effects. This includes stimulating the maturation and activation of DCs and macrophages, enhancing the production of granzymes and perforins in CTLs and NK cells (70, 71), and bolstering the proportion of memory T cells (72, 73). Activated NK cells secrete cytokines such as IFN-γ and TNF-α, which synergistically activate DC cells, fostering robust proliferation of CD8^+^ T cells. Notably, the abundant IFN-γ produced by NK cells induces high expression of mbIL-15 in a subset of bystander DC cells, thereby promoting antigen-specific CD8^+^ T-cell responses and eliciting adaptive immunity against cancer cells (74, 75).

Moreover, type I IFNs directly facilitate the proliferation of CD8^+^ T cells and the differentiation of T-helper (TH)-1 cells while suppressing TH2 cell development (76–78). Thus, among innate cytokines, type I IFNs emerge as crucial signals that shape the repertoires of effector and memory T cells.

Evidence from studies, such as that conducted by Steven Lohard et al., highlights the synergistic effect of type I IFNs and TNF-α in inducing enhanced apoptosis in breast cancer recipient cells, underscoring the multifaceted role of these factors in promoting antitumor responses (52).

### 2.3 cGAS-STING and immune cells dynamics

A myriad of immune cells, including NK cells, CD4^+^ T cells, CD8^+^ T cells, DCs, and macrophages, respond to the antineoplastic effects induced by STING activation (41). Activation of STING in endothelial cells within the TME may contribute to tumor vascular remodeling, positively influencing tumor regression (71, 79). DCs are highly proficient APCs. DCs, as proficient APCs, tend to engulf tumor-derived DNA or cGAMP, subsequently inducing type I IFN production, which relies on STING presence (65, 80, 81). These DCs serve as a significant source of type I IFNs and TNF-α, both contributing to the promotion of an inflammatory TME (52). Moreover, tumor-derived cGAMP triggers a STING-mediated interferon response in non-tumor cells and activates NK cells, mediating the clearance of CD8^+^ T cell-resistant tumors in response to STING agonists, underscoring the broad applicability of STING activation in promoting antitumor responses across various tumor contexts (70, 71, 82, 83).

Tumors exhibiting cGAS positivity are characterized by elevated concentrations of T-cell-derived effector cytokines, such as IFN-γ and TNF-α, and demonstrate enhanced responsiveness to ICIs, indicative of a “hot” tumor phenotype (65). Notably, CD8^+^ T cells are predominantly found in regions expressing cGAS, suggesting a direct role for cGAS in driving CD8^+^ T cell infiltration, especially in tumors with heterogeneous cGAS expression (65). Additionally, the absence of cGAS expression in cancer cells, coupled with its presence in adjacent non-diseased tissues, implies a potential immune evasion mechanism (65).

Activation of the cGAS-STING cascade supports the maintenance of stem cell-like properties post T-cell differentiation (84). In xenograft models, STING agonists facilitate the generation of stem cell-like central memory CD8^+^ T cells, augmenting the antitumor response to chimeric antigen receptor (CAR) T cell therapy in cancer patients (84). Moreover, in triple-negative breast cancer (TNBC) with BRCA-deficiency, PARP inhibitor therapy relies on the recruitment of CD8^+^ T cells, mediated through the intratumoral STING pathway (85, 86). Furthermore, intratumoral administration of exogenous cGAMP, which enhances STING activity, significantly enhances anti-tumor CD8^+^ T cell responses, effectively controlling both injected and contralateral tumors (87).

### 2.4 Remodeling the cold tumor microenvironment via STING signaling

Activation of the STING pathway initiates a transformative process within the cold TME, leading to significant tumor regression and heightened immunity (41, 88). In a study focusing on STING agonists like DMXAA, conducted on murine models with cold pancreatic tumors, researchers observed a remarkable alteration in the immunologic landscape of the TME, resulting in prolonged survival rates (89, 90). Specifically, within the tumor, there was a notable increase in inflammatory chemokines and maturation markers of DCs and CTLs (89).

Furthermore, researchers identified a gain-of-function mutation variant of STING, known as STINGN^153S^, near the STING dimer site, extracted from patients with autoimmune diseases (19). Introducing this persistently active STING signal into cancer cells led to robust expression of chemokines, attracting infiltrating immune cells, particularly CD8^+^ T cells and NK cells, into the activated TME (19).

Moreover, studies have demonstrated that intratumoral injection of cGAMP, a second messenger of the STING pathway, induces potent antitumor responses by promoting the accumulation of activated macrophages within the TME, a process contingent on STING activation (91).

## 3 cGAS-STING related cancer therapy research areas

### 3.1 STING agonists

STING agonists have emerged as promising candidates for enhancing immune cell recruitment in the TME. They can be categorized into several classes, including cyclic dinucleotides (CDNs), small molecule agonists, bacterial carriers, agonists for *in vivo* delivery of antibody-coupled drugs, and nanovaccines (92). Vaccines utilizing STING agonists are developed based on the mechanism of the cGAS-STING pathway (93). In therapeutic cancer vaccines, CDNs act as effective adjuvants, robustly stimulating immune responses against tumors. This activation involves the activation of CTLs and NK cells, leading to consistent regression observed in various mouse tumor models (94). Direct intratumoral administration of selected CDNs in mouse models of B16 melanoma, CT26 colon cancer, and 4T1 breast cancer has resulted in rapid and significant tumor regression, with no apparent local or systemic toxicity. This intervention promotes persistent systemic T cell immunity targeting the relevant antigens (88). The wide-ranging potential applications and remarkable safety profile of CDNs have led to the initiation of clinical trials for STING agonists (**Table 1**). DMXAA, a mouse STING agonist, demonstrated strong antitumor activity in mice but failed in clinical trials due to its inability to activate human STING (95).

**Table 1:** Clinical trials of STING agonists.

Drug name	Trial number	Therapy area	Combination Therapies	Route	Highest status	Active companies	Target-based actions	Extract
XMT-2056	NCT05514717	Advanced/recurrent solid tumors that express HER2	Monotherapy	i.v.	Phase 1 Clinical (Recruiting)	Mersana Therapeutics Inc.	Erbb2 tyrosine kinase receptor modulator; STING stimulator	Antibody drug conjugate targeting HER2 antigens with STING agonist
TAK-500	NCT05070247	Select locally advanced or metastatic solid tumors	Monotherapy or with Pembrolizumab	i.v.	Phase 1 Clinical (Recruiting)	Takeda Pharmaceutical Co. Ltd.	STING stimulator	Antibody drug conjugate with STING agonist
SYNB1891	NCT04167137	Select locally advanced or metastatic solid tumors	Monotherapy or with Pembrolizumab	i.t.	Phase 1 Clinical (Completed)	Synlogic Inc.	STING stimulator	*Escherichia coli* expressing dacA protein
CDK-002 (exoSTING)	NCT04592484	Subjects With Advanced/Metastatic, Recurrent, Injectable Solid Tumors	Monotherapy	i.t.	Phase 2 Clinical (Completed)	Codiak BioSciences Inc.	STING stimulator	Intraluminal delivery of CDN STING agonists in exosomes
ADU-S100	NCT02675439	Advanced/Metastatic Solid Tumors or Lymphomas	Monotherapy or in combination with Ipilimumab	i.t.	Phase 2 Clinical (Terminated)	Chinook Therapeutics Canada Inc.	STING stimulator	Small molecule of CDN
ADU-S100	NCT03937141	PD-L1 positive recurrent or metastatic HNSCC	With Pembrolizumab	i.t.	Phase 2 Clinical (Terminated)	Chinook Therapeutics Canada Inc.	STING stimulator	Small molecule of CDN
MIW815 (ADU-S100)	NCT03172936	Advanced/Metastatic Solid Tumors or Lymphomas	With the PD-1 checkpoint inhibitor PDR001	i.t.	Phase 1 Clinical (Terminated)	Novartis Pharmaceuticals	STING stimulator	Small molecule of CDN
IMSA-101	NCT05846659	Oligoprogressive Solid Tumor Malignancies	With immune checkpoint inhibitor (ICI) Immunotherapy (PULSAR-ICI)	i.t.	Phase 2 Clinical (Recruiting)	Genor Biopharma Co. Ltd. ImmuneSensor Therapeutics Inc.	STING stimulator	Small molecule of CDN
Ulevostinag (MK-1454)	NCT03010176	Advanced/metastatic solid tumors or lymphomas	With Pembrolizumab	i.t.	Phase 2 Clinical (Completed)	Merck Sharp & Dohme Corp.	STING stimulator	Small molecule of CDN
BI-STING (BI 1387446)	NCT04147234	Advanced cancer (solid tumors)	Monotherapy or with BI 754091 (anti–PD-1 monoclonal antibody)	i.t.	Phase 1 Clinical (Active, not recruiting)	Boehringer Ingelheim International GmbH	STING stimulator	Small molecule of CDN
BMS-986301	NCT03956680	Advanced Solid Cancers	Monotherapy or with Nivolumab and Ipilimumab	i.t./i.m.	Phase 1 Clinical (Active, not recruiting)	Bristol-Myers Squibb Co.	STING stimulator	Small molecule of CDN
E-7766	NCT04144140	Advanced solid tumors or lymphomas	Monotherapy	i.t.	Phase 1 Clinical (Terminated)	Eisai Inc.	STING stimulator	Small molecule of CDN
SB-11285	NCT04096638	Advanced solid tumors	Monotherapy or with atezolizumab	i.v.	Phase 1 Clinical (Recruiting)	F-star Therapeutics Ltd.	STING stimulator	Small molecule of CDN
TAK-676	NCT04420884	Advanced solid tumors	Monotherapy or With Pembrolizumab	i.v.	Phase 1 Clinical (Recruiting)	Takeda Oncology	STING stimulator	Small molecule of CDN
GSK-3745417	NCT03843359	Advanced Solid Tumors	Monotherapy or with Dostarlimab	i.v.	Phase 1 Clinical (Active, not recruiting)	GSK plc	STING stimulator	Nonnucleotide small molecule
HG-381	NCT04998422	Advanced solid tumors	Monotherapy	i.v.	Phase 1 Clinical (Recruiting)	HitGen Ltd.	STING stimulator	Nonnucleotide small molecule
SNX-281	NCT04609579	Advanced solid tumors and lymphoma	Monotherapy or with Pembrolizumab	i.v.	Phase 1 Clinical (Terminated)	Silicon Therapeutics	STING stimulator	Nonnucleotide small molecule
MK-2118	NCT03249792	Advanced/metastatic solid tumors or lymphomas	Monotherapy or with Pembrolizumab	i.t./s.c.	Phase 2 Clinical (Completed)	Merck Sharp	STING stimulator	Non-CDN small molecule

i.t., intratumorally; i.v., intravenously; i.m., intramuscular injection; s.c., subcutaneously.Searched through ClinicalTrials.gov on April 2, 2024.

ADU-S100 (MIW815), a synthetic analogue of 2’,3’-cGAMP, an endogenous agonist, has been engineered to enhance its half-life, ensuring consistent activation of the STING pathway and highlighting its therapeutic potential (96). Outcomes from a Phase I clinical trial (NCT02675439) demonstrated the well-tolerated nature of MIW815 in patients with advanced or metastatic cancers, with an overall response rate (ORR) of 2.1% as monotherapy. Treatment-related adverse effects included fever, chills, injection site discomfort, and headache. Although the therapeutic efficacy of MIW815 as a standalone treatment appeared limited, indications of systemic immune activation have emerged, such as increased plasma cytokine levels and systemic T-cell clonal expansion (97). In a Phase Ib clinical trial (NCT03172936) investigating the safety and tolerability of combining MIW815 with spartalizumab in patients diagnosed with advanced/metastatic solid tumors or lymphomas, the combined therapy showed good tolerance. However, the observed ORR of 10.4% demonstrated mild anti-tumor effects (98).

MK-1454, a synthetic CDNs analog developed by Merck & Co., has been designed for the management of advanced or metastatic solid tumors and lymphoma. Initial findings from a phase I clinical trial (NCT03010176) showed that while MK-1454 monotherapy led to an increase in the release of pro-inflammatory cytokines, it did not result in a complete or partial response within the monotherapy cohort (n=20) (96). However, when administered in combination with pembrolizumab, the treatment achieved an ORR of 24%, accompanied by a median reduction of 83% in target tumors. Both the monotherapy and combination therapy cohorts reported treatment-related adverse events (TRAEs) at rates of 82.6% and 82.1%, respectively (96, 99). Although the study concluded in April 2022, the final results are still pending.

SYNB1891 is an engineered live strain capable of producing CDNs under hypoxic conditions to activate the STING pathway. Findings from the study NCT04167137 indicated that repeated intratumoral administration of SYNB1891, either alone or in combination with Atezolizumab, exhibited favorable safety and tolerability, along with evidence of activation of specific targets within the STING pathway (100). Concurrently, the study documented five cases of cytokine release syndrome in the monotherapy treatment group (100).

Mn^2+^ itself is a potent cGAS activator that increases the sensitivity of the DNA sensor cGAS and its downstream junction protein STING, which induces cellular production of type I IFN and cytokines in the absence of any infection *in vivo* (101). Mn^2+^ was found to significantly facilitate DC and macrophage maturation and antigen presentation in a cGAS-STING-dependent manner, enhance CD8^+^ T cell and NK cell activation, and increase the number of CD44^hi^CD8^+^ T cells. Mn^2+^ significantly promotes antitumor immunotherapy across diverse mouse models, and a phase 1 clinical trial completed in patients with advanced metastatic solid tumors yielded promising evidence affirming well-tolerated safety profile and expected antitumor effects of Mn^2+^ in patients. The clinical trial (NCT03991559) enrolled 22 patients with advanced metastatic solid tumors, and phase I clinical data demonstrated 45.5% (95% CI, 26.9-65.3) best objective response and 90.9% (95% CI, 72.2-97.5) best disease control rate. All 5 patients who had failed prior PD-1 therapy in combination with chemotherapy or radiotherapy demonstrated disease control, including three experiencing partial response (PR) while two maintained stable disease (SD) (95). Of these patients, 86% experienced any grade of treatment-related adverse event (ae), and 41% experienced serious treatment-related adverse events (grades 3-4). Grades 1-2 adverse events were well tolerated, while grades 3-4 adverse events were managed with supportive care measures. Additionally, five patients with widespread abdominal metastases developed acute suspicious local or systemic cytokine release syndrome (CRS). The simple and stable composition of Mn^2+^, the low cost, and wide accessibility of Mn^2+^ render this therapy appealing and highly assuring, and it is currently undergoing phase II clinical trials (95).

### 3.2 Radiotherapy and chemotherapy

Radiotherapy, as a first-line treatment for many solid tumors, exerts its cytotoxic effects mainly by inducing DNA damage in tumor cells (102–105). In addition to this, radiation therapy can also participate in anti-tumor activity by modulating the tumor microenvironment and promoting anti-tumor immune responses, one mechanism of which involves the excitation of the cGAS- STING pathway (105–107). Radiation therapy leads to accumulation of dsDNA in cancer cells, which can be monitored by cGAS as a cytosolic DNA sensor (108), leading to activation of cGAS/STING signaling. An optimal radiation dose could effectively activate the cGAS-STING signal pathway to prompt the production of IFN-β. Research demonstrated that administering nanoparticle-cGAMP through inhalation alongside radiotherapy induced deterioration of lung metastases through an effector CD8^+^ T-cell approach mediated by the STING signaling pathway (62, 109). The aforementioned results suggest that the antitumor immunity produced by radiotherapy alone or radiotherapy in combination with STING agonists is dependent on the modulation of CD8^+^ T-cell activation and differentiation by the cGAS-STING signaling pathway, and also point towards a potential usage of cGAS-STING signaling pathway agonists in conjunction with radiotherapy in oncology treatment (62). Clinical trial NCT03538314 is currently evaluating the impact of treatment through modulation of the cGAS-STING pathway on response to radiotherapy and ICIs for the treatment of patients with advanced solid tumors.

Chemotherapy induces DNA damage while inhibiting DNA repair. Damaged DNA activates cGAS-STING to enhance DC-mediated antigen presentation and T cell responses (44, 105, 110). For example, one of the mechanisms of cisplatin treatment is the initiation of the cGAS/STING pathway by upregulating the protein levels of cGAS and STING (53). The ability of the topoisomerase I inhibitor topotecan to restrict the proliferation of breast cancer cells *in vivo* was eliminated in mice deficient in STING (111). In addition, plethora of studies have indicated that cisplatin in combination with IFN, not only impede tumor growth effectively but also extend survival time in mice, compared with cisplatin alone (112–114). 5-Fluorouracil (5-FU) is a crucial chemotherapeutic agent that is predominantly involved in disrupting the DNA synthesis. Researchers found that the combination of cGAMP and 5-FU further enhanced the anti-tumor immune response elicited by 5-FU and effectively reduced the cytotoxic effects associated with 5-FU. The mechanism likely entails cGAMP activating the STING signaling pathway, subsequently inducing cytokine production activating DC. Activated DC not only induces cross priming of CD8^+^ T cells to generate anti-tumor responses, but also seems to reduce 5-FU-induced cytotoxicity (115). These outcomes suggest that chemotherapeutic agents may also elicit antitumor effects by activating the cGAS-STING signaling pathway, and that the combination of chemotherapeutic agents with agonists of the cGAS-STING signaling pathway represents a fruitful area of research in oncology therapy (62). Clinical trial NCT03410901 aims to investigate the efficacy and safety of the combination of cGAS-STING pathway activators and chemotherapy in patients with advanced solid tumors.

### 3.3 IR (irradiation)+ ATRI effectively induces activation of classical versus non-classical cGAS-STING signaling pathways

The study demonstrates that combining ATR inhibitors with radiotherapy effectively enhances the activation of both classical and non-classical cGAS-STING signaling pathways. Specifically, the addition of ATR inhibitors to radiotherapy significantly boosts STING signaling in mouse models of colorectal cancer, leading to heightened innate immunity and increased infiltration of TILs. This phenomenon has the potential to remodel the TME and enhance the efficacy of ICIs in anti-ICI tumors. During the investigation, inhibition of STING in CT26 cells resulted in a notable decrease in infiltrating CD8^+^ cells and levels of IFNs following treatment with IR+ ATRI, indicating that the immunomodulatory effects of IR+ ATRI are contingent upon STING signaling (52, 60).

### 3.4 G4 Binders

G-4 conjoined (G4s or G-quadruplexes) are unconventional nucleic acid configurations integral to biological mechanisms (116). They represent potential anticancer targets, with the pursuit of effective G4 binders allowing for the identification of cytotoxic ligands capable of interfering with selective G4 structures on oncogenes or telomeres. Notably, G4 binders such as pyridostigmine and PhenDC3 have been observed to elicit several effects, including an increase in micronuclei, activation of the cGAS-STING pathway in both human and mouse cancer cells, production of type I interferons, and activation of intrinsic immunity (116).

One such G-quadruplex stabilizer is CX-5461 (117) which induces severe cancer cell death by obstructing replication forks or generating single-stranded DNA gaps or breaks, particularly in cancer cells lacking functional homologous recombination (HR) and non-homologous end joining (NHEJ) mechanisms (117–119). CX-5461 initiates IRF-3-mediated type I interferon expression by activating the cGAS-STING pathway. Researchers have noted elevated levels of cGAMP and active interferon regulatory factor 3 (IRF3) in colorectal cancer (CRC) cells (118), followed by an increase in type I interferon, which triggers downstream signaling, including JAK1/TYK2-mediated phosphorylation of signal transducer and activator of transcription 1 (STAT1) on tyrosine residue 701 (Tyr701) to form STAT1-STAT1 homodimers (118). These dimers then translocate to the nucleus and bind to the interferon-γ activating site (GAS), initiating the transcription of numerous genes, including programmed death-ligand 1 (PD-L1), thereby mediating PD-L1 expression to indirectly kill CRC cells (120–122).

Furthermore, administration of CX-5461 alone or in combination with anti-PD-1 has been shown to augment cytotoxic T lymphocyte (CTL) count, while reducing the number of bone MDSCs in the spleen of mice (123). Therefore, the cGAS-STING pathway plays a crucial role in G4 conjoined-related drugs, and various drugs have the potential to convert “cold” tumors into “hot” tumors, providing a potential approach for combined immunotherapy in immune-resistant tumors to enhance the efficacy of ICI response (118).

### 3.5 Anti-cancer effects exerted by inhibition of the cGAS-STING pathway

The anti-cancer effects resulting from inhibition of the cGAS-STING pathway have garnered increasing attention in recent research. While previous studies have primarily focused on the anti-tumor effects of STING agonists, emerging evidence suggests that STING inhibitors also possess inhibitory effects on tumor activity. Li et al. demonstrated that tumor CIN modifies the TME and stimulates the cGAS-STING pathway (124). Specifically, in the presence of a functional immune system, chromosomally unstable tumor cells activate STING, triggering ER stress and promoting tumor cell metastasis (125–127). CIN, prevalent in advanced cancers, disrupts the normal cell division process, contributing significantly to cancer’s treatment resistance, spread, and metastasis (128, 129). The study introduced a novel technique called Contact Tracing for single-cell RNA sequencing data analysis, revealing that chronic CIN-induced activation of the cGAS-STING pathway fosters downstream signaling reconnection in cancer cells, thereby establishing a pro-metastatic TME. This reconnection involves a rapid type I IFNs response selectively mediated by STING, along with an associated increase in ER stress response from cancer cells. Conversely, reversing CIN, eliminating cancer cell STING, or inhibiting ER stress response signaling abolished the TME-dependent effects of CIN reversal and suppressed tumor metastasis in immunocompetent environments, although these interventions had no effect in severely immunocompromised environments (124).

Similarly, Sirui et al. reported comparable findings, indicating that STING may impede the anti-cancer capabilities of NK cells through the IRF3-induced regulatory B cells (Bregs) pathway, contributing to the limited efficacy of STING monotherapy (130). This study identified the STING/IRF3/IL-35 signaling axis as the underlying cause of the limited efficacy of STING agonists, leading to immune evasion by inducing Bregs to secrete IL-35, independent of type I IFNs. Combining STING agonists with anti-IL-35 treatments showed promising efficacy in preclinical models of pancreatic and lung cancer, offering a new avenue for tumor immunotherapy (99). As a pivotal signal transduction molecule involved in the intrinsic immune response *in vivo*, STING significantly regulates type I IFNs and influences the body’s anti-tumor immune response (55). While STING agonists have been extensively studied and hold high expectations in tumor immunotherapy, the consistent lack of success in related studies suggests the presence of a potential negative immunomodulatory mechanism associated with STING activation.

## 4 Discussion

Epigenetic suppression of cGAS in cancer cells curtails the innate immune response to cytoplasmic DNA accumulation, fostering immune evasion (23, 83). Luis A. Martinez et al. elucidated that WTp53 facilitates the degradation of the cytoplasmic DNA exonuclease TREX1, leading to cytoplasmic DNA buildup and subsequent activation of the cGAS/STING pathway. Their findings imply a novel mechanism through which the tumor suppressor TP53 exploits the pathogen recognition receptor cGAS to suppress tumors, thereby activating the innate immune response to impede tumor growth. Elevated cytoplasmic DNA levels in cancer cells are a hallmark, contributing to a persistent basal activation of the cGAS/STING pathway, which can potentially be induced (45, 55, 131). Conversely, evidence suggests that in genetically unstable cells, the cGAS/STING pathway may facilitate metastatic progression. However, in this specific context, the activation of IRF3 appears notably absent (45, 125). The precise mechanism responsible for the attenuation of the signaling pathway to IRF3 from cGAS/STING, despite the existence of cytoplasmic DNA in tumor cells, remains poorly understood (45). Studies have demonstrated that Mtp53 disrupts the functionality of the cytoplasmic DNA sensing machinery, particularly the cGAS-STING-TBK1-IRF3 pathway, crucial for initiating the innate immune response. By interacting with TBK1, Mtp53 (but not wildtype p53) impedes the assembly of the trimeric complex involving TBK1-STING-IRF3, essential for IRF3 activation, nuclear translocation, and subsequent transcriptional activity (132). The restoration of TBK1 function holds promise for reactivating immunosurveillance and eliminating mtp53 tumors (132).

In addition to genomic instability, factors such as the release of heterologous DNA and mitochondrial damage caused by pathogen invasion and replication can lead to abnormal leakage of dsDNA within the body. This dsDNA is then recognized by the DNA receptor cGAS, consequently activating the cGAS-STING signaling pathway (41). Therefore, the cGAS-STING mechanism constitutes a comprehensive surveillance system in response to tissue damage and pathogen invasion, as abnormalities in this mechanism can result in infection (41). Stringent regulatory mechanisms are paramount to uphold cellular and tissue homeostasis under normal conditions owing to the non-specified activity of cGAS on DNA. SPSB3 has been identified as a cGAS-targeted substrate receptor that binds to the cullin-RING ubiquitin ligase 5 (CRL5) complex, facilitating the attachment of ubiquitin to nuclear cGAS (133). Apart from removing nucleosome-bound cGAS, SPSB3 may also assist in disassembling DNA from cGAS. This broad binding capacity and the irreversible nature of the degradation process contribute to an efficient mechanism for cGAS inactivation. This study elucidates the mechanism by which the ubiquitin proteasome system (UPS) degrades nuclear cGAS in circulating cells (133). It has been demonstrated that the binding of the MRE11-RAD50-NBN complex to nucleosome fragments is necessary to enable the release of cGAS from acid patch-mediated segregation, facilitating its mobilization and activation by dsDNA. Consequently, MRE11 is crucial for cGAS activation in response to oncogenic stress, cytoplasmic dsDNA, and ionizing radiation (134).

In clinical trials, the anticipated efficacy observed in mouse models for combating tumors was not replicated, except with Manganese. Based on the relevant literature, we have consolidated viewpoints regarding key aspects: 1. Age Factor: While age was tightly controlled in experimental mouse models, clinical trials, including NCT02675439, involved participants with a diverse age range, with a median age of 61 (94, 97). The response to vaccine administration may decline with advancing age (135, 136). Consequently, similar to many vaccines, the adjuvant potential of CDN vaccines is negatively affected by the aging process (137). Future animal research should explore the influence of mouse age on the efficacy of CDN vaccines and utilize mice that reflect the target age demographic, which may yield more precise experimental data for guiding clinical trials. 2. Tumor Heterogeneity: Current clinical trials include diverse patient cohorts with advanced/metastatic solid tumors or lymphomas, representing over ten distinct cancer types, such as melanoma, breast cancer, sarcoma, pancreatic cancer, etc. (96–98, 100). The varied immune profiles within different TMEs, along with variations in the genomic presence of the STING pathway, inherently impact immune modulation and clinical responses (97). Additionally, differences in tumor types may result in fluctuations in injection pressures during intratumoral delivery, causing inconsistencies in drug exposure and potentially influencing experimental outcomes (97). 3. Disparities in the STING gene: The human STING gene exhibits significant heterogeneity and shows population stratification (138, 139). There are over five allele variants of the human STING gene, including R232 (wild-type), H232, HAQ (R71H-G230A-R293Q), AQ (G230A-R293Q), and Q293, with a population frequency exceeding 1% (94). Notably, the HAQ-STING variant is prevalent in East Asian populations but rare in Africa, while the AQ and Q293 variants are exclusive to African populations (138, 139). The presence of these STING allele variants within the population can significantly impact the response to CDN vaccines (94) Substantial evidence suggests compromised functionality of the HAQ and H232 allele variants in STING (94). Therefore, the development of a CDN capable of effectively activating H232 or HAQ-STING seems essential (94). In the mentioned clinical trials, there was a significant range of ethnicities among the participants, including Black, Caucasian, Asian, and others (97). 4. Drug Resistance: It is crucial to acknowledge that the participants in current clinical trials primarily consist of individuals with advanced cancer who have experienced disease progression after undergoing multiple lines of therapies. This trend contributes to a heightened prevalence of multi-drug resistance, and the presence of unexplained heterogeneity poses challenges in effectively demonstrating the efficacy of STING agonists. 5. Drug Concentration: The need for repeated tissue sampling in participants has resulted in insufficient pharmacokinetic data for intratumoral injections (NCT02675439). Consequently, it remains uncertain whether the concentration of locally injected MIW815 corresponds to the concentration required for activity, as observed in preclinical models (97). Therefore, further research is needed to refine pathways for delivering drugs to the TME. Gathering relevant administration data, such as injection techniques, tumor tissue pressure, drug residence time at the injection site, and duration of tumor tissue exposure, could be crucial for future investigations into the intratumoral administration of small molecule immune agonists (97). Additionally, it is important to recognize that while most clinical trials have reported favorable evaluations regarding the safety and tolerability of STING agonists (96–98, 100), instances of serious treatment-related adverse reactions, including cytokine release syndrome, have been documented (100). The central concern associated with immune stimulatory therapy lies in the potential for a “cytokine storm,” as prolonged activation of STING can result in excessive cytokine production, leading to severe toxicity and potential fatality. However, the clinical understanding of how to effectively terminate the activated signaling to prevent this remains limited (96).

The clinical efficacy of STING agonists is constrained by several factors, including inadequate cytoplasmic delivery, swift immune clearance, lack of specific cellular targeting, and systemic inflammatory responses (140). The progression in STING agonist development has reached a critical impasse, akin to many contemporary immunotherapeutic agents (140). Consequently, the current studies have pivoted towards the utilization of varied vectors for STING agonist administration (140). The advent of nanotechnology offers a promising avenue for targeted delivery to specific tissues and cells, enhancing the absorption of immunomodulatory agents. This approach is anticipated to bolster cancer immunotherapy while mitigating adverse effects. Current research into lipid-based nanostructures for STING agonist conveyance is expanding, with Hao et al. assessing the capacity of different lipid nanoparticles (NPs) to amplify the impact of STING agonist treatments (140). Liposomal formulations of STING agonists, when used in conjunction with therapies such as radiotherapy, chemotherapy, vaccines, monoclonal antibodies (mAbs), or immune checkpoint inhibitors (ICIs), demonstrate significant potential in eliciting synergistic therapeutic effects (140). Li et al. have elaborated on a novel tumor microenvironment-responsive DNA-based nanomedicine encapsulated within dendritic mesoporous organosilica nanoparticles (DMONs). This formulation, as a standalone treatment, yielded a tumor growth inhibition (TGI) rate of 51.0% in the murine B16-F10 melanoma model. Furthermore, when combined with an anti-PD-L1 antibody, it significantly bolstered anti-tumor efficacy, culminating in near-total tumor elimination (141). Antibody Drug Conjugate (ADC) is a complex consisting of a cytotoxic drug attached to a tumor-targeting monoclonal antibody, which combines the characteristics of a targeted drug with those of a chemotherapeutic drug to achieve precision therapy. Applying the ADC strategy to the development of STING agonism, Merck Sharp & Dohme developed a STING ADC drug, XMT-205614, an ADC platform for STING agonists that employs a potent acyclic dinucleotide STING agonist, a cleavable ester-based linker, and a hydrophilic PEG8 bisglucosamine scaffold (142, 143). Tumor-targeted ADCs constructed using the resulting STING agonist platform have potent and long-lasting antitumor activity and exhibit high stability and good pharmacokinetics in non-clinical species. α epidermal growth factor receptor (EGFR)-172 ADC is a tumor-targeted ADC constructed with the cGAMP analog IMSA172 linked to EGFR antibody via a cleavable Mc-Val-Cit-PABC linker. EGFR antibody to generate ADCs, which showed potent antitumor efficacy in a synthetic mouse tumor model and exhibited good tolerability (144). It not only has further synergistic effects with anti-PD-L1 antibodies to achieve superior anti-tumor efficacy but also promotes multiple aspects of innate and adaptive anti-tumor immune responses. Mixing this small molecule ADC drug with human serum for up to 24 hours does not reduce its activity, and the payload IMSA172 also shows good stability in the same serum for 24 hours, which is not comparable to cGAMP drugs. STING ADC drugs have become the direction of development in the field of STING (144).

Furthermore, a series of studies have highlighted that the cGAS-STING signaling pathway promotes tumor growth. For instance, in cases where DSBs occur within cancer cells, cGAS relocates to the nucleus, thereby impeding the formation of PARP1–TIMELESS complexes, which inhibit homologous recombination (HR) repair and sustain CIN, thereby facilitating tumor evolution. Research has also indicated that upon recognition of CIN, cGAS activates non-canonical NF-κB, contributing to a program that enhances tumor metastasis (125). Treatments involving mutated 7,12-dimethylbenzanthracene (DMBA), cisplatin, and etoposide have been reported to facilitate skin carcinogenesis by triggering inflammatory cytokines and phagocytes that are STING-dependent (53). In the context of brain metastases, cyclic GMP-AMPs (cGAMPs) that metastasize to neighboring cells, such as astrocytes, can lead to the production of IFN-β and TNF-α within the TME. However, in this particular scenario, the response tends to promote tumor growth and contribute to resistance to chemotherapy (145). A deep understanding of the dual role of STING pathway activation in halting cancer progression is imperative. Clearer treatment protocols and the identification of selective biomarkers for specific tumor types, which can predict the therapeutic benefits of STING activation, are urgently needed (140). Benguigui et al. recently identified a novel biomarker: interferon-stimulated Ly6E^hi^ neutrophils, whose presence in both mice and human blood strongly correlates with successful outcomes in immunotherapy across various cancer types (146). These neutrophils are generated through tumor-specific activation of the STING pathway and can sensitize tumors unresponsive to anti-PD1 therapy, partly via IL12b-mediated activation of cytotoxic T cells (146). Wang et al. have developed PolySTING nanoparticles, incorporating cGAMP within a micelle nanoparticle formed from the STING-activating polymer polyethylene oxide-b-PSC7A (147). Employing a ‘shock-and-lock’ dual activation approach, they demonstrated that conventional type I dendritic cells (cDC1) are crucial for the STING-mediated elimination of established and metastatic murine tumors (147). Through multiplex immunohistochemistry analysis, they categorized non-small cell lung cancer patients into groups with high and low STING-activating cDC1 levels. Patients with high cDC1 activation showed prolonged progression-free and overall survival rates in both neoadjuvant chemotherapy and neoadjuvant chemotherapy plus Pembrolizumab settings, highlighting the potential of STING-activating cDC1 signatures (XCR1^+^STING^+^CXCL9^+^) as prognostic biomarkers for therapy response (147). In personalized medicine, predictive biomarkers are vital for tailoring treatments to individual patients and their specific tumor characteristics, yet reliable biomarkers for immune checkpoint inhibitor outcomes across different cancer types are still needed (146).

The mucosal immune system (MIS), or mucosa-associated lymphoid tissue (MALT), comprises immune tissues, cells, and molecules within the mucosal linings of the respiratory, gastrointestinal, and genitourinary tracts, exerting a critical function in the immune defense. The mucosal immunity pathway equips T cells with a mucosal homing program, enhancing their recruitment to initial activation sites (148). Evidence suggests that mucosal immunity more effectively induces resident memory T cells (Trm) at mucosal tumor locations compared to traditional systemic administration (149, 150). These Trm cells, a subset of long-lived memory T cells, reside in tissues, particularly at mucosal surfaces exposed to the environment, and do not recirculate (151). Intranasal vaccination with a mucosal vector in a head and neck cancer model showed induced local Trm and tumor suppression, correlating high Trm levels with improved overall survival in lung cancer patients (151). Thus, inducing persistent Trm is proposed as a novel target for developing effective therapeutic cancer vaccines (151). Manganese (Mn^2+^), a robust stimulator of type I interferon, activates the cGAS-STING pathway even without infection, showcasing its potential in immunotherapy. Mn^2+^ is actively and efficiently transported into cells via diverse transporters, such as divalent metal transporter (DMT1), Zn^2+^ and Ca^2+^ transporters (152, 153). Nasally administered Mn^2+^ produces immune responses akin to those triggered by cholera toxin B, including prolonged induction of IgA antibodies in lungs, saliva, and serum (154). The antitumor effects of Mn^2+^ stem from its promotion of monocyte-derived DC differentiation, DC maturation, and antigen presentation, alongside chemokine production that facilitates immune cell recruitment and germinal center formation, heavily reliant on the cGAS-STING pathway’s bridging role between innate and adaptive immunity (154). Research using a melanoma model in mice indicated that CD103^+^ dendritic cells (DCs) are unique in delivering intact antigens to tumor-draining lymph nodes, activating tumor-specific CD8^+^ T cells (155). Enhancing the myeloid lineage commitment to CD103^+^ DCs and their activation within tumors can significantly improve responses to checkpoint and BRAF inhibitors (155). Furthermore, the efficacy of intratumoral CD8^+^ T cell activation is linked to the presence of CD103^+^ DCs, suggesting their key role in overcoming limited T cell infiltration in certain tumors (156). Consequently, mucosal delivery emerges as a promising vaccine administration route, necessitating innovative formulation strategies for broader application.

## 5 Conclusion

The synergistic potential of combining the cGAS-STING signaling pathway with ICI therapy in the treatment of malignant tumors presents a promising avenue for clinical translation. However, before the successful integration of STING agonists into clinical practice can be achieved, several challenges must be addressed. Similar to the inflammation induced by cGAS-STING, inflammatory responses against tumors stimulated by cGAS-STING may display time sensitivity. While transient activation of cGAS-STING within innate immune cells could enhance antitumor efficacy, prolonged activation may lead to immune tolerance (81), thereby promoting tumor progression. Consequently, the impact of cGAS-STING on cancer outcomes depends on various factors, including tumor type, the host’s immune profile, specific cell types activated, therapeutic approach employed, and the degree of cGAS-STING activation. Further research is crucial to define the precise role of cGAS-STING in oncology and to elucidate both its distinct benefits and potential drawbacks associated with targeting the cGAS-STING pathway in cancer therapeutics.

## Author contributions

MH: Methodology, Visualization, Writing – original draft, Writing – review & editing, Supervision, Validation. ZC: Supervision, Validation, Writing – original draft, Writing – review & editing. RL: Data curation, Writing – original draft. ML: Software, Writing – review & editing. NG: Supervision, Writing – review & editing. TK: Validation, Writing – review & editing. FG: Funding acquisition, Resources, Writing – original draft, Writing – review & editing. WC: Conceptualization, Writing – review & editing, Funding acquisition, Writing – original draft.
